# Minimum carbon dioxide is a key predictor of the respiratory health of pigs in climate-controlled housing systems

**DOI:** 10.1186/s40813-024-00408-3

**Published:** 2024-12-20

**Authors:** Eddiemar Baguio Lagua, Hong-Seok Mun, Keiven Mark Bigtasin Ampode, Hae-Rang Park, Md Sharifuzzaman, Md Kamrul Hasan, Young-Hwa Kim, Chul-Ju Yang

**Affiliations:** 1 Animal Nutrition and Feed Science Laboratory, Department of Animal Science and Technology, Sunchon National University, Suncheon, 57922 Republic of Korea; 2 Department of Multimedia Engineering, Sunchon National University, Suncheon, 57922 Republic of Korea; 3 Department of Animal Science, College of Agriculture, Sultan Kudarat State University, Tacurong City, 9800 Philippines; 4 Department of Animal Science and Veterinary Medicine, Bangabandhu Sheikh Mujibur Rahman Science and Technology University, Gopalganj, 8100 Bangladesh; 5 Department of Poultry Science, Sylhet Agricultural University, Sylhet, 3100 Bangladesh; 6 Interdisciplinary Program in IT-Bio Convergence System (BK21 Plus), Chonnam National University, Gwangju, 61186 Republic of Korea; 7 Interdisciplinary Program in IT-Bio Convergence System (BK21 Plus), Sunchon National University, 255, Jungangno, Suncheon, 57922 Republic of Korea

**Keywords:** Environmental stress, Animal welfare, Health management, Swine smart farming, Artificial intelligence

## Abstract

**Background:**

Respiratory disease is an economically important disease in the swine industry. Housing air quality control is crucial for maintaining the respiratory health of pigs. However, maintaining air quality is a limitation of current housing systems. This study evaluated the growth and health parameters of pigs raised under different environmental conditions and identified key environmental variables that determine respiratory health. Eighty (Largewhite × Landrace) × Duroc crossed growing pigs (31.71 ± 0.53 kg) were equally distributed into two identical climate-controlled houses with distinct environmental conditions (CON = normal conditions and TRT = poor conditions). Two-sample tests were performed to compare the means of the groups, and a random forest algorithm was used to identify the importance scores of the environmental variables to respiratory health.

**Results:**

Pigs in the TRT group were significantly exposed to high temperatures (28.44 vs 22.78 °C, *p* < 0.001), humidity (88.27 vs 61.86%, *p* < 0.001), CO_2_ (2,739.93 vs 847.91 ppm, *p* < 0.001), NH_3_ (20.53 vs 8.18 ppm, *p* < 0.001), and H_2_S (14.28 vs 6.70 ppm, *p* < 0.001). Chronic exposure to these factors significantly reduced daily feed intake (1.82 vs 2.32 kg, *p* = 0.002), resulting in a significant reduction in average daily gain (0.72 vs 0.92 kg, *p* = 0.026), increased oxidative stress index (3.24 vs 1.43, *p* = 0.001), reduced cortisol levels (2.23 vs 4.07 mmol/L, *p* = 0.034), and deteriorated respiratory health status (74.41 vs 97.55, *p* < 0.001). Furthermore, a random forest model identified Min CO_2_, Min NH_3_, and Avg CO_2_ as the best predictors of respiratory health, and CO_2_ was strongly correlated with NH_3_ and H_2_S concentrations.

**Conclusions:**

These findings emphasize the critical importance of proper environmental management in pig farming and suggest that regular monitoring and control of either CO_2_ or NH_3_, facilitated by environmental sensors and integration into intelligent systems, can serve as an effective strategy for improving respiratory health management in pigs.

**Supplementary Information:**

The online version contains supplementary material available at 10.1186/s40813-024-00408-3.

## Background

The implementation of climate-controlled housing systems in pig production enhances the productivity of farms by providing a better environment promoting better health conditions, faster growth rates, and a better feed conversion ratio in fattening pigs but also higher fertility in breeder pigs than in pigs in naturally ventilated housing systems [1–3]. However, despite advancements in housing systems, seasonal variations in performance are still observed in pigs [4, 5] and poultry [6, 7]. This occurred because the variations in the environmental conditions inside the house are influenced by the external environment, primarily temperature [8, 9]. Therefore, the environmental factors inside the house are not fully controlled in the current housing system. A typical climate-controlled house is equipped with a temperature-based ventilation system that has a cooling system and a heating system, primarily to maintain the temperature inside the house [10]. However, it has some limitations in controlling humidity in hot humid seasons or climates [11] and toxic gases during cold seasons in temperate climates [8, 9, 12]. During the cold season, airflow is automatically reduced to maintain the temperature inside the house. However, with limited air exchange, pigs are at risk of accumulating toxic gases such as carbon dioxide (CO_2_), ammonia (NH_3_), and hydrogen sulfide (H_2_S) produced through pigs’ respiration and urine and feces excretion and from decomposing organic matter in the slurry pit or bedding [13, 14]. These factors have adverse effects on the biology of pigs.

Respiratory disease is one of the most economically important diseases in pigs. It is caused by several pathogenic bacteria and viruses and is induced by elevated levels of certain environmental factors [15]. NH_3_ and H_2_S are the most health-threatening gases in pig farms to both humans and pigs [16, 17]. High levels of NH_3_ can irritate the respiratory tract, resulting in increased respiratory health symptoms such as coughing and sneezing [18, 19], and chronic exposure even at 15 ppm in growing pigs can induce oxidative stress and immune system suppression and alter the nasal bacterial population, favoring pathogenic bacterial growth and leading to respiratory infection [20]. On the other hand, H_2_S is a highly toxic gas second only to cyanide, which has respiratory and nervous system toxicological effects [21, 22]. Chronic exposures below 10 ppm or as low as 0.03 ppm have been reported to be associated with nasal, ocular, respiratory, and neurological effects in humans [21], and the same conditions were observed in pigs at low concentrations [23]. Although CO_2_ is the least toxic among the three gases, its concentration is strongly associated with the NH_3_ and H_2_S concentrations [17]. Therefore, the control of either one of the gases can potentially control other gases to a minimum. In addition to ventilation, several approaches, such as diet manipulation to limit nitrogen and sulfur excretion [24–26], the use of feed additives to inhibit the production or to bind toxic gases to inhibit volatilization [27, 28], slurry treatment, such as acidification to reduce bacterial activity [29], and frequent removal of slurry or bedding [30], are used to manage these toxic gas concentrations inside the house. The combination of these is the best approach, but the cost of the operation must be considered.

Toxic gas concentrations can be controlled to minimum levels through the integration of gas sensors into ventilation systems or into automatic slurry or litter management systems. With this system, the concentrations of toxic gases are automatically controlled, enabling better health management for both pigs and humans. However, adding several parameters to the system adds complexity to the feedback control system of the ventilation system. Nevertheless, advancements in computer science can manage these complexities by providing advanced and robust algorithms. Alternatively, a simple but robust system is possible by integrating only the key environmental factors significant to pigs' health. Fortunately, continuous detection and monitoring of the respiratory health conditions of pigs is possible with the help of artificial intelligence (AI) with high accuracy [15]. The availability of AI technology and environmental sensors provides detailed information on the animals and their environment. Furthermore, subtle relationships between environmental factors and animal conditions can be further understood via machine learning techniques. In this study, the growth performance and health parameters of growing pigs raised under different environmental conditions were evaluated, and a machine learning algorithm was used to identify key environmental variables that determine respiratory health.

## Materials and methods

### 2.1 Experimental design

A total of 80 healthy growing (Largewhite × Landrace) × Duroc crossed pigs with similar weights (31.71 ± 0.53 kg) were selected for this study. Two identical climate-controlled houses at the Sunchon National University swine experimental farm were used. The houses were divided into four pens (2.35 × 2.90 m) with fully slatted plastic floors and equipped with a heating and cooling system (BUW1450M9S, LG Electronics, South Korea) and three mechanical exhaust fans (EURO-500S, Euro Housing Co., Ltd., South Korea) automatically controlled by a temperature-based ventilation system (Euro Housing Co., Ltd., South Korea). Ten pigs with similar weights were equally distributed in each pen. One house was used for the control (CON) group, where the ventilation system was set up to maintain environmental conditions ideal for growing pigs. The second house was used for the treatment (TRT) group. In this house, poor environmental conditions were simulated by setting the fans’ speed to no more than 50% of their capacity to achieve an atmospheric NH_3_ level of at least 20 ppm. However, the desired NH_3_ level was not achieved at the start of the growing period. Slurry manure was added 1 week before the commencement of the study, and the slurry was maintained until the end of the growing period in the treatment facility. The pigs were grown for 21 days during the winter season from the 8th to the 29th of December 2023.

The stocking density was 0.60 m^2^/head, excluding feeding and water systems and other equipment. An automatic wet‒dry feeder (LFS-120, IONTECH Co., Ltd., South Korea) and a water trough were installed per pen, and the feeders were refilled every afternoon. The pigs had ad libitum access to feed and water, and the houses were illuminated artificially 24 h a day throughout the 21-day growing period. No medication was used during the study period. This study received approval from the institutional review board and ethics committee of Sunchon National University, South Korea (SCNU IACUC-2023-19).

### 2.2 Environmental conditions

**Fig. 1:**
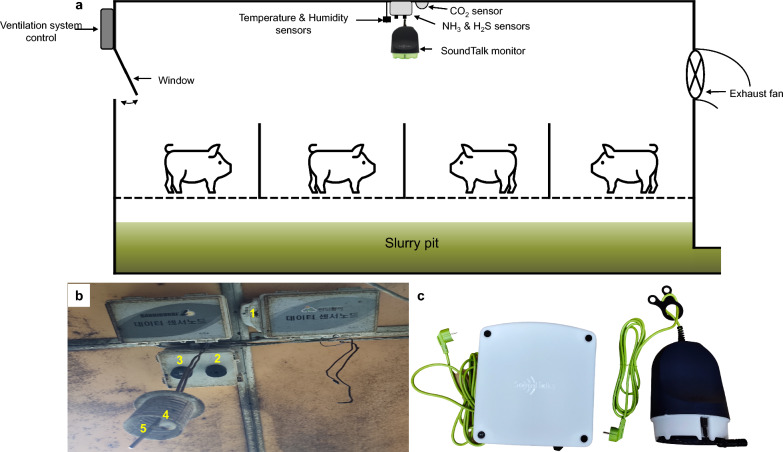
Environmental and animal sensors: **a** Illustration of the experimental house, showing the locations of the environmental and animal sensors; **b** the carbon dioxide, ammonia, hydrogen sulfide, humidity, and temperature sensors, labeled 1–5, respectively; and **c**) SoundTalk (SoundTalks NV, Leuven, Belgium) display (left) and monitor (right)

An environment monitoring system (Farm Note, Nare Trends Inc., South Korea) was used for automatic detection and monitoring of environmental parameters such as temperature, relative humidity, CO_2_, NH_3_, and H_2_S (Fig. 1a and b). The electronic sensors were installed by the manufacturer in the middle of the house 2 m from the floor. The environmental data were logged at 5-min intervals and stored and retrieved from the server.

### Growth performance parameters

The individual body weights of the pigs were measured in the morning at a consistent time each week. The remaining feeds were manually collected from the feeder bins and weighed, and fresh feeds were provided after body weight data collection. The feed intake is the difference between the total amount of feed given and the remaining feed amount. Body weight gain (BWG), average daily gain (ADG), and the feed conversion ratio (FCR) were calculated using the equations below.

$$BWG \left( {kg} \right) = Final\, Body\, Weight\, \left( {kg} \right) - Initial\, Body\, Weight\left( {kg} \right)$$

$$ADG \left( {kg/day} \right) = \frac{{Body\, Weight\, Gain \left( {kg} \right) }}{{Growing\, Period \left( {days} \right)}}$$

$$FCR = \frac{{Feed\, Intake \left( {kg} \right)}}{{Body\, Weight\, Gain \left( {kg} \right)}}$$

### Blood biochemical parameters

On the last day of the growing period, 3 pigs from each replicate or 12 pigs per group were randomly selected to determine the blood NH_3_, blood urea nitrogen (BUN), lactate dehydrogenase (LDH), aspartate aminotransferase (AST), total oxidant status (TOS), total antioxidant status (TAS), oxidative stress index (OSI), and cortisol levels. The OSI is the ratio of TOS to TAS multiplied by 100 [31]. The pigs were snared, and blood samples were collected from the cervical vein via a disposable syringe. At least 5 ml of blood was collected and transferred into a serum separator vacuum tube (SSTTM II Advance BD Vacutainer, Becton and Dickinson and Company, United Kingdom). The blood samples were kept in a Styrofoam box with ice and immediately sent to the laboratory for centrifugation at 3000 rpm for 15 min. Serum samples were collected, placed in microcentrifuge tubes, and then stored at − 24 °C until analysis. The serum samples were sent to an external laboratory for analysis.

### Respiratory health

The respiratory health status of each herd was automatically evaluated using an artificial intelligence (SoundTalks NV, Leuven, Belgium), which is composed of two devices, a monitor and a gateway (Fig. 1c). The monitor is the sensory device of SoundTalks. It has temperature, humidity, and sound sensors (only the sound data from this device were used for the analysis). It can collect sounds within a 10-m radius inside a house. All the data collected are sent wirelessly to the gateway. The gateway receives all the data collected from one or more monitors within a 30-m radius. It has an LAN connection, and it sends the data to the SoundTalks cloud where data processing occurs [15]. One SoundTalk gateway was installed between the two houses, and one monitor was installed in the middle of each house following the manufacturer’s guidelines. Coughing sounds are detected and quantified from the collected sound data and transformed into a metric (0–100) on the basis of a proprietary algorithm that represents the respiratory health status (ReHS) score of the herd. A high ReHS score indicates a high respiratory health status. According to the manufacturer’s manual, a yellow warning is notified once the ReHS score falls below 60 to 40, which indicates potential respiratory problems. A ReHS under 40 is a red warning that indicates a high risk of respiratory problems. All the collected data were stored and accessed online (https://www.soundtalksweb.com).

### Data preprocessing

The environmental data were preprocessed prior to further analysis. There was no missing data; however, outliers were identified. The outliers were identified via Z score statistics, with values exceeding ± 3 standard deviations considered extreme. To address these outliers, a percentile-based approach was employed. Specifically, extremely high values, defined as those with *Z* scores greater than 3, were replaced with the 99th percentile value, whereas extremely low values (*Z* scores less than − 3) were substituted with the 1st percentile value. This method ensures that outlier values are replaced with representative values derived from the data distribution.

$$Z = \frac{{\left( {X - \mu } \right)}}{\sigma }$$

Here, *Z* is the calculated Z score of the data point; *X* is the data point; *μ* is the mean of the variable; and *σ* is the standard deviation of the variable.

### Model training and variable importance

Five internal house environmental parameters relevant to pig health, such as temperature, humidity, CO_2_, NH_3_, and H_2_S, were collected. Pigs were exposed to fluctuating levels of these environmental parameters throughout the day and the growing period. While conventional practice often relies on assessing pig conditions on the basis solely of average parameter values, this study adopts a more comprehensive approach. In this investigation, the significance of these environmental parameters concerning ReHS was evaluated using not only the average (Avg) values but also the minimum (Min) and maximum (Max) values. This enables a thorough examination of the range of environmental conditions experienced by pigs.

The random forest (RF) algorithm is a popular machine learning algorithm used for classification and regression [32]. It constructs an ensemble of decision trees, each trained on a random subset of data and features at each split. Each tree is diverse, which improves the overall performance of the model. The final prediction is made by aggregating the predictions of all individual trees. Feature importance scores are computed based on how much each feature reduces impurity across all trees in the forest. These scores indicate the relative importance of each feature in predicting the independent variable. In the current study, an RF was used to identify the importance scores of the environmental values concerning ReHS. The training and evaluation of the RF models and the extraction and ranking of importance scores were conducted in RStudio using the `caret` and `randomforest` packages. The dataset was split into training (70%) and testing (30%) subsets before model training. The model was trained with tenfold cross-validation to optimize model performance. The combinations of variables of the models are shown in Additional file 1.

### Statistical analyses

RStudio version 4.3.1 was used for the statistical analyses. All the data were tested for normality of distribution using the Shapiro‒Wilk test and for homogeneity of variance using Levene’s test. Parameters with significance (*p* < 0.05) in either of the tests were subjected to the Mann‒Whitney U test to compare significant differences between groups. Otherwise, the two-sample t test was employed. The significance level was set at *p* < 0.05. Descriptive statistics are provided in the additional files.

The correlations between ReHS and the environmental parameters were calculated with Pearson correlation analysis. Separately, the correlation of blood biochemical parameters was also assessed using the same test. A significant correlation is set at the < 0.05 significance level.

The models were evaluated with the root-mean-square error (*RMSE*) and coefficient of determination (*R*^2^) using the equations shown below.

$$RMSE = \sqrt {\frac{1}{n}\mathop \sum \limits_{i = 1}^{n} \left( {y_{i} - \hat{y}_{i} } \right)^{2} }$$

$$R^{2} = 1 - \frac{{\mathop \sum \nolimits_{i = 1}^{n} \left( {y_{i} - \hat{y}_{i} } \right)^{2} }}{{\mathop \sum \nolimits_{i = 1}^{n} \left( {y_{i} - \overline{y}} \right)^{2} }}$$

where *n* is the number of observations; ${y}_{i}$ is the actual value for the *i*-th observation; ${\widehat{y}}_{i}$ is the predicted value for the *i*-th observation; and ∑ denotes the summation of all observations from *i* = 1.

The model with the lowest *RMSE* and *R*^*2*^ closer to 1 is considered the model with the best prediction performance.

## Results

### Environmental conditions

**Fig. 2:**
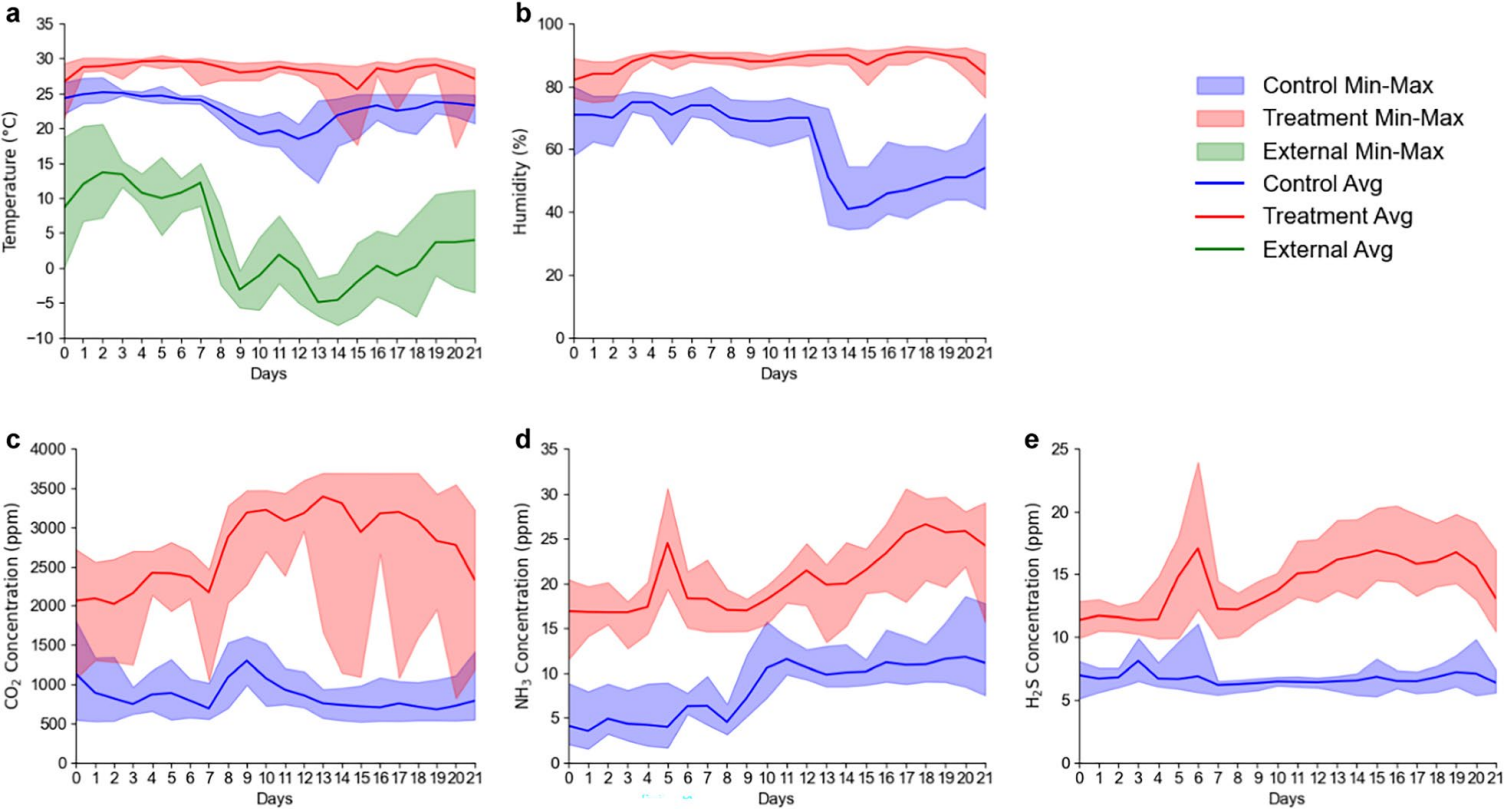
Daily environmental conditions in the CON and TRT groups: **a** temperature, **b** humidity, **c** carbon dioxide, **d** ammonia, and **e** hydrogen sulfide concentrations

The descriptive statistics of the environmental parameters are shown in Additional file 2, and the trends are illustrated in Fig. 2. The differences in all the environmental parameters were highly significant (*p* < 0.001) with increasing temperature (22.78 vs 28.44 °C), humidity (61.86 vs 87.27%), CO_2_ (847.91 vs 2,739.93 ppm), NH_3_ (8.18 vs 20.53 ppm), and H_2_S (6.70 vs 14.28 ppm) in the TRT group. The lowest recorded temperature was 12.17 °C in the CON group and 17.18 °C in the TRT group during the second half of the growing period. During this period, the temperature fluctuations were considerable as the external temperature began to drop below zero. Additionally, humidity was reduced in the CON group but remained high in the TRT group during the same period.

The CO_2_ levels in the TRT group fluctuated more than those in the CON group, and those in the TRT group tended to increase but were almost stable in the CON group. The highest recorded CO_2_ concentration in the TRT group was 3,693 ppm, and the lowest was 820 ppm, which was similar to the average CO_2_ concentration in the CON group. There was an increasing trend in NH_3_ levels in both groups. The highest level in the CON group was 18.56 ppm, which was recorded on the 20th day. The NH_3_ and H_2_S levels in the TRT group reached 30.60 and 24.00 ppm, respectively. The highest H_2_S level recorded in the CON group was 12.09 ppm.

### Growth performance

**Fig. 3:**
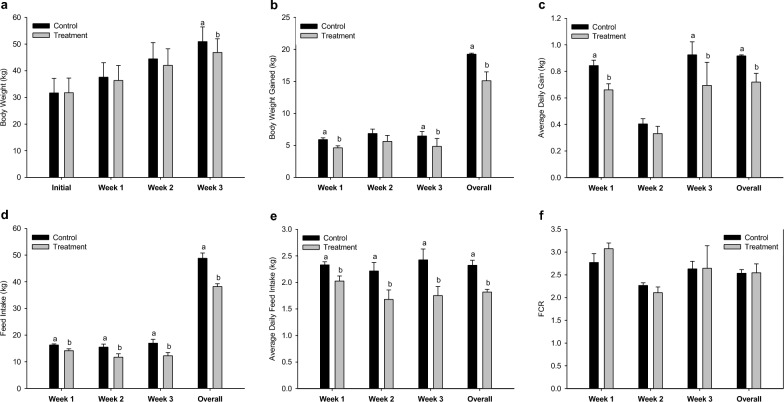
Weekly and overall growth performance of pigs under different environmental conditions: **a** body weight, **b** body weight gained, **c** average daily gain, **d** feed intake, **e** average daily feed intake, and **f** FCR. Means with different superscripts (a & b) are significantly different (*p* < 0.05)

The weekly and overall growth performance of the pigs are shown in Fig. 3 (see Additional file 3). The body weight of the CON group was significantly greater than that of the TRT group in week 3 (50.92 vs 46.85, *p* = 0.021). Body weight gain and average daily gain were significantly (*p* < 0.05) different in the 1st week, 3rd week, and overall. The differences in feed intake and average daily feed intake between the CON and TRT groups were significant (*p* < 0.05) and increased over time throughout the growing periods. The overall average daily feed intake of the CON group was 2.32 kg, which was significantly (*p* = 0.002) greater than that of the TRT group (1.82 kg). However, the FCR was not negatively affected (2.54 vs 2.55, *p* = 0.830).

### Blood biochemical parameters

As shown in Table 1, pigs in the TRT group presented higher concentrations of blood NH_3_ (190.56 vs 151.89 µmol/L), AST (45.78 vs 34.11 IU/L), and LDH (627.11 vs 562.89 U/L) than those in the CON group. However, these differences were not statistically significant (*p* > 0.05). Additionally, no significant change in the BUN concentration was observed, but the BUN concentration in the CON group was greater than that in the TRT group (7.11 vs. 6.56 mmol/L, *p* = 0.527). Compared with the CON group, the TRT group presented significantly greater TOS (21.58 vs 6.52 µmol/L, *p* < 0.001) and OSI (32.4 vs 1.43 mmol/L, *p* = 0.001), indicating oxidative stress. Surprisingly, the cortisol concentrations were significantly greater in the CON group than in the TRT group (4.07 vs 2.23 µg/dL, *p* = 0.034). Pearson’s correlation analysis revealed high AST and LDH concentrations with increasing blood NH_3_ (Table 2).

**Table 1:** Respiratory health status and blood biochemical parameters of pigs under different environmental conditions

Parameters	Control	Treatment	SEM^1^	*P* value
ReHS	97.55^a^	74.41^b^	2.23	< 0.001
BUN mmol/L	7.11	6.56	0.42	0.527
Blood NH_3_ µmol/L	151.89	190.56	11.74	0.387
AST U/L	34.11	45.78	6.30	0.863
LDH U/L	562.89	627.11	61.96	0.796
TOS µmol/L	6.52^b^	21.58^a^	2.34	< 0.001
TAS mmol/L	0.47^b^	0.69^a^	0.05	0.024
OSI	1.43^b^	3.24^a^	0.32	0.001
Cortisol mmol/L	4.07^a^	2.23^b^	0.44	0.034

^1^Standard error of the mean; *BUN* blood urea; *NH*_*3*_ ammonia; *AST* aspartate aminotransferase; *LDH* lactate dehydrogenase; *TOS* total oxidant status; *TAS* total antioxidant status; *OSI* oxidative stress index. Means with different superscripts (a & b) are significantly different (*p* < 0.05)

**Table 2:** Pearson’s correlation analysis of blood biochemical parameters

	BUN	Blood NH_3_	AST	LDH	Cortisol
BUN					
Blood NH_3_			0.892**	0.899**	
AST		0.892**		0.931**	
LDH		0.899**	0.931**		
Cortisol					

*BUN* blood urea*; NH*_*3*_ ammonia*; AST* aspartate aminotransferase*; LDH* lactate dehydrogenase*.* ** = *p* < 0.01

### Respiratory health status

**Fig. 4:**
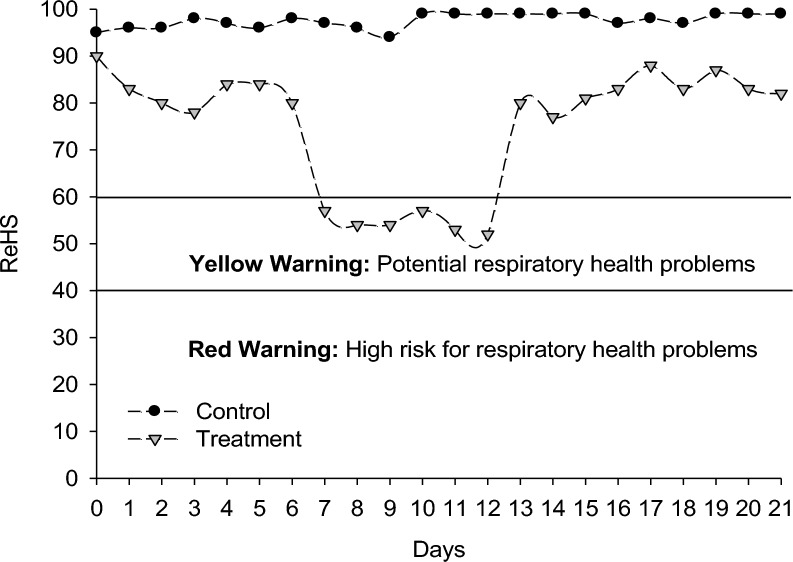
Daily respiratory health status (ReHS) of pigs under different environmental conditions

ReHS was significantly (*p* < 0.001) affected by changes in environmental conditions (Table 1 and Fig. 4). The pigs in the TRT group had an average ReHS of 74.41, whereas those in the CON group had an average of 97.55. The ReHS in the CON group was stable and close to 100, indicating good respiratory health conditions. A significant reduction in the ReHS score from the 7th day until the 12th day was observed in the TRT group, and during these periods, yellow warnings from SoundTalks were issued, indicating potential respiratory health problems. The ReHS scores increased and stabilized at the same levels as those observed in the first week from the 13th day until the end of the growing period. However, the ReHS scores remained lower than those of the CON group.

### Key indicators of respiratory health

**Fig. 5:**
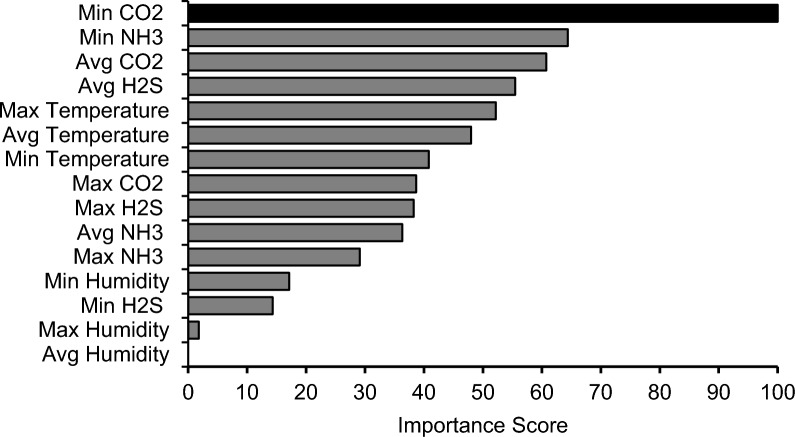
Importance scores of different environmental parameter values to the respiratory health status of pigs

**Fig. 6:**
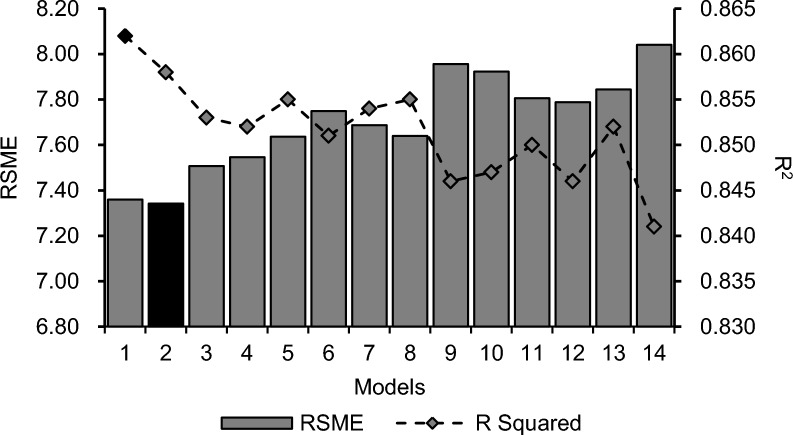
Performance of the random forest models in predicting the respiratory health status of pigs

Figure 5 shows the level of importance of each of the environmental parameters affecting the ReHS of the pigs. Min CO_2_ had the highest importance, with an importance score of 100. It was followed by Min NH_3_, Avg CO_2_, Avg H_2_S, and Max Temperature, with importance scores of 64.40, 60.77, 55.46, and 52.18, respectively. The four least important variables were Avg, Max, and Min Humidity (0, 1.80, and 17.14, respectively), and Min H_2_S (14.32). The performance of the models is shown in Fig. 6, which reveals that the combination of Min CO_2_ and Min NH_3_ had the highest *R*^*2*^ (0.862). However, the addition of Avg CO_2_ to the model improved the *RMSE* (7.34) of the model. However, the differences in *R*^*2*^ and *RMSE* between models 1 and 2 were small. Furthermore, the prediction performance decreases with the addition of other variables.

**Fig. 7:**
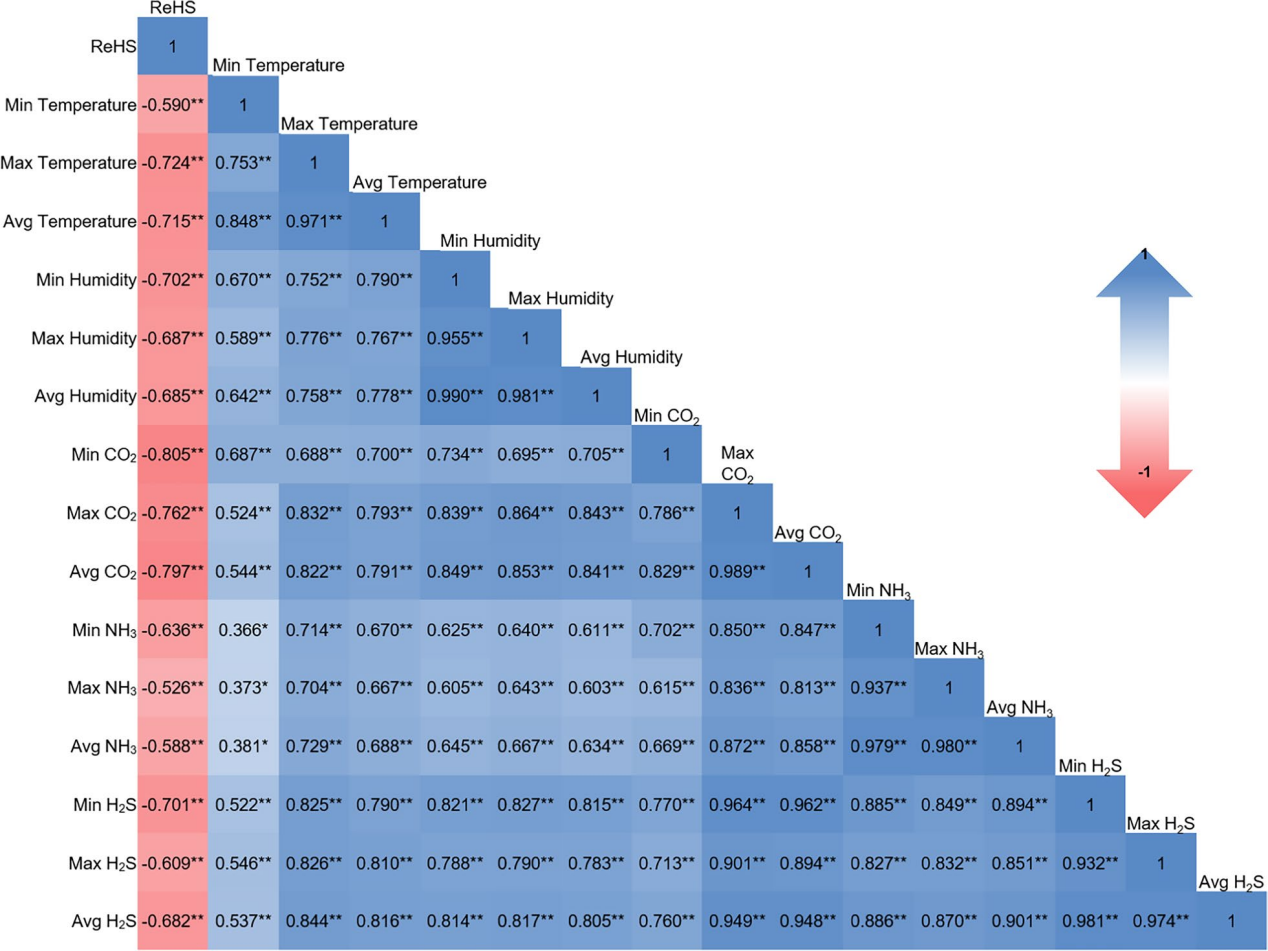
Pearson’s correlation analysis of respiratory health status and different environmental parameter values (* = *p* < 0.05, ** = *p* < 0.01)

The results of the Pearson’s correlation analysis revealed that all the environmental variables presented significant (*p* < 0.05) and at least moderate negative (*r* > − 0.50) correlations with the ReHS, as shown in Fig. 7. The ReHS was strongly negatively correlated with Min, Avg, and Max CO_2_ (*r* = -0.805, -0.797, and -0.762, respectively); Max and Avg temperature (*r* = -0.724 and -0.715, respectively); Min humidity (*r* = − 0.702); and Min H_2_S (*r* = − 0.701). Furthermore, moderate (*r* > 0.50) to very strong (*r* > 0.900) positive correlations between independent variables were observed. Min CO_2_ had strong correlations with Min NH_3_ and all H_2_S values. However, the Avg and Max CO_2_ values were very strongly correlated with the other toxic gases’ Min, Max, and Avg values.

## Discussion

The concentrations of toxic gases in the current study were higher than the Min, Max, and Avg concentrations of NH₃ and CO₂ recorded in both the CON and TRT groups in our previous study conducted during the summer [10]. This increase is likely a result of reduced ventilation rates to maintain the indoor temperature during colder seasons in a temperature-based ventilation system [9, 12]. Reduced air exchange leads to the accumulation of toxic gases.

Pigs are exposed to fluctuating levels of environmental parameters and, if not controlled, can have a significant effect on their growth and health [20, 33, 34]. These findings are demonstrated in the current study, where the feed intake of the pigs was restricted by elevated temperature, humidity, and the presence of toxic gases such as CO_2_, NH_3_, and H_2_S inside the house, resulting in a significant decrease in growth performance. The TRT group had 21.73% less feed intake than the CON group, which reduced the average daily gain by 21.51% without affecting feed efficiency. A reduction in feed intake is one of the thermoregulatory responses of pigs during heat stress [35], but it can also be observed when they are exposed to at least 15 ppm NH_3_ [20], at least 7.18 ppm H_2_S [33], or very high CO_2_ (40,000 ppm) [36]. However, the CO_2_ concentrations in the current study did not reach that level. The differences in feed intake increased over time, suggesting that the degree of effects of the environmental stressors differed across the different stages of the pigs and the length of exposure.

BUN is a waste product formed in the liver from the breakdown of excess protein [37]. In this study, the reduced BUN in the TRT group could be due to reduced protein intake resulting from a reduction in feed intake. NH_3_ and H_2_S are the most important air pollutants on farms because of their toxic effects [16, 17, 24]. Consistent with the results of the current study, Wang et al. [20] reported an increase in blood NH_3_ in pigs exposed to increasing atmospheric NH_3_ and induced oxidative stress. Additionally, high ambient temperature and high H_2_S and CO_2_ concentrations are known to induce reactive oxygen species (ROS) production, leading to oxidative stress in animals [38–40]. Pigs in the TRT group were apparently under oxidative stress, as shown by their high TOS and OSI values. The increase in TAS was the response of the pigs to eliminate oxidants in the body, however, the OSI indicates that the antioxidant system was overwhelmed by high oxidants. Additionally, the difference in TOS between the CON and TRT groups was too high, which may indicate the synergism of the abovementioned environmental factors. However, the effects of each parameter and their interactions cannot be quantified as a limitation of the experimental design in the current study. These gases coexist in the pig house; therefore, there is a need to study their interactions to determine their minimum threshold levels when they coexist. Increased oxidants in the body lead to oxidative damage to cellular components, including proteins, lipids, and DNA. This damage can disrupt cell membranes, organelles, and other cellular structures, leading to cell injury or death [41–43]. This can be attributed to the increased blood AST and LDH concentrations in the TRT group, as these enzymes leak into the bloodstream from damaged cells.

The production of cortisol is a response to animals experiencing stress, leading to a spike in cortisol levels [44]. However, in chronic conditions, the hypothalamic‒pituitary‒adrenal (HPA) axis, which controls the stress response, can become dysregulated under prolonged stress [45]; this is the reason for the low cortisol level observed in the TRT group, which is consistent with the study of O’Connor et al. [46]. This mechanism protects the body from the adverse effects of elevated cortisol, which are not limited to inflammation, muscle breakdown, or immune suppression [45, 47].

AI has been applied to monitor respiratory health in pigs [15], poultry [48], and cattle [49]. In the current study, respiratory health was automatically monitored using AI, which revealed that pigs exposed to poor environmental conditions had low ReHS. Additionally, the alarm system of the AI was triggered from day 7 until 12, which is indicative of respiratory distress. NH_3_ and H_2_S can irritate mucous membranes in the eyes and respiratory tracts of pigs, and prolonged exposure to these gases can damage the respiratory tract and impair the immune response, increasing the susceptibility of pigs to respiratory infections [20, 21]. Furthermore, high ambient temperature and high atmospheric CO_2_ can exacerbate the toxic effects of NH_3_ and H_2_S by increasing the respiration rate and volume [38], leading to greater exposure to toxic gases. Additionally, high humidity can exacerbate heat stress effects and respiratory distress in pigs by increasing the solubility of NH_3_ and H_2_S in the air [50, 51], creating a favorable environment for pathogen growth [52], and increasing mucous membranes in the respiratory tract, increasing their sensitivity to NH_3_ and H_2_S, which can increase respiratory distress symptoms [53].

As discussed above, environmental control is crucial for optimizing growth and maintaining the respiratory health of pigs, and many studies have associated NH_3_ with respiratory disease [10, 19, 54]. However, average values were used in previous studies, other toxic gases were not considered, and the levels of environmental factors changed over time. In the current study, RF analysis revealed that Min CO_2_ had the greatest importance score on the ReHS of pigs and was highly correlated. Although CO_2_ is not very potent in inducing respiratory distress, its concentrations are strongly positively associated with the concentrations of more potent gases, such as NH_3_ and H_2_S, since these gases are natural products of microbial activity on organic material in slurry pits and bedding. Specifically, NH_3_ and H_2_S are produced from the decomposition of nitrogenous compounds (e.g., proteins and amino acids) and sulfur-containing compounds (e.g., methionine and cysteine), respectively, and during these processes, CO_2_ is also released [55]. Additionally, urease-producing bacteria can hydrolyze urea excreted in urine into NH_3_ and CO_2_ [56]. In the study of Peng et al. [51], CO_2_ was also found to have the highest importance in predicting the NH_3_ concentration in pig houses, as much of the NH_3_ results from the breakdown of proteins and urea, processes in which CO_2_ is also produced, as previously described. These findings suggest that respiratory health could be indirectly improved by controlling the CO_2_ concentration, which may, in turn, help regulate NH_3_ and H_2_S concentrations, both of which are known as respiratory stressors in the housing environment.

The combination of Min CO_2_, Min NH_3_, and Avg CO_2_ produced the best model in terms of *RSME* to predict ReHS in pigs. However, to simplify the model, the Min CO_2_ and Min NH_3_ combination can be used since the *RSME* difference between the two models was small (7.36 vs 7.34), and it had the highest *R*^*2*^ value (0.862 vs 0.858). Adding more variables to the model deteriorates the model *R*^*2*^ and prediction performance. This could be due to adding complexity to the model and the multicollinearity of the variables. Figure 7 shows that the variables had collinearity. Highly correlated variables add redundant information, which does not improve the model and can lead to unstable estimates [57, 58]. However, the RF can address multicollinearity [59]. Respiratory disease is caused by multiple factors that are not limited to environmental factors. Nevertheless, machine learning models can be useful in identifying and selecting the best predictors of respiratory disease that can be used as tools to improve the health management of pig farms.

The limitations of this study include the absence of ventilation rate data, which is also an important factor in respiratory health. Including ventilation rate could provide deeper insights into environmental influences on respiratory health of pigs. Another limitation was that the AI tool used generated only one ReHS score per day. We recommend the use of an alternative AI model capable of generating high-resolution temporal data on respiratory distress symptoms (e.g., coughing and sneezing), with values recorded at frequent intervals (e.g., every 5 min or hourly) rather than once daily. This increased data granularity would allow for a more precise linkage with environmental factors recorded simultaneously, thereby enhancing the validation and robustness of the findings of the current study.

## Conclusion

The results of the current study revealed that environmental stress significantly reduced the growth performance, respiratory health, and overall health parameters of growing pigs. The tissue and metabolic indicators were numerically increased and exhibited significantly lower cortisol levels in pigs with long-term exposure to poor environments. The analysis revealed that minimum CO_2_ and minimum NH_3_ values are key indicators of pigs’ respiratory health. These findings provide a valuable reference for building models to predict respiratory health based on environmental parameters. Additionally, the findings suggest that integrating CO_2_, NH_3_, or both into intelligent environmental control systems can potentially improve the management of pigs' respiratory health.

## Supplementary Information

Additional file 1.

Additional file 2.

Additional file 3.

## Data Availability

The datasets used and/or analyzed during the current study are available from the corresponding author and the first authors upon reasonable request.

## Abbreviations

ADG: Average daily gain
AST: Aspartate aminotransferase
Avg: Average
BUN: Blood urea nitrogen
BWG: Body weight gain
CO_2_: Carbon dioxide
CON: Control
FCR: Feed conversion ratio
H_2_S: Hydrogen sulfide
HPA: Hypothalamic‒pituitary‒adrenal
LDH: Lactate dehydrogenase
Max: Maximum
Min: Minimum
NH_3_: Ammonia
OSI: Oxidative stress index
R^2^: Coefficient of determination
ReHS: Respiratory health status
RF: Random forest
RMSE: Root-mean-square error
TAS: Total antioxidant status
TOS: Total oxidant status
TRT: Treatment
